# GPS tracking analyses reveal finely-tuned shorebird space use and movement patterns throughout the non-breeding season in high-latitude austral intertidal areas

**DOI:** 10.1186/s40462-023-00411-3

**Published:** 2023-09-01

**Authors:** Enzo Basso, Johannes Horstmann, Eldar Rakhimberdiev, José M. Abad-Gómez, José A. Masero, Jorge S. Gutiérrez, Jorge Valenzuela, Jorge Ruiz, Juan G. Navedo

**Affiliations:** 1 Bird Ecology Lab, Instituto de Ciencias Marinas y Limnológicas, Universidad Austral de Chile, Valdivia, Chile; 2 Programa de Doctorado en Ecología y Evolución, Facultad de Ciencias, Universidad Austral de Chile, Valdivia, Chile; 3 Department of Theoretical and Computational Ecology, Institute for Biodiversity and Ecosystem Dynamics, University of Amsterdam, Amsterdam, The Netherlands; 4 Department of Anatomy, Cell Biology and Zoology, Faculty of Sciences, University of Extremadura, Badajoz, Spain; 5 Ecology in the Anthropocene, Associated Unit CSIC-UEX, Zoology, Faculty of Sciences, University of Extremadura, Badajoz, Spain; 6 Centro de Estudios y Conservación del Patrimonio Natural (CECPAN), Chiloé, Chile; 7 Estación Experimental Quempillén, Facultad de Ciencias, Universidad Austral de Chile, Chiloé, Chile; 8 Millennium Institute Biodiversity of Antarctic and Subantarctic Ecosystems (BASE), Santiago, Chile

**Keywords:** Circadian rhythms, Continuous-time stochastic process, Long-distance migration, Movement ecology, Nomadism, Range-residency

## Abstract

**Background:**

Long-distance migratory birds spend most of their annual cycle in non-breeding areas. During this period birds must meet their daily nutritional needs and acquire additional energy intake to deal with future events of the annual cycle. Therefore, patterns of space use and movement may emerge as an efficient strategy to maintain a trade-off between acquisition and conservation of energy during the non-breeding season. However, there is still a paucity of research addressing this issue, especially in trans-hemispheric migratory birds.

**Methods:**

Using GPS-tracking data and a recently developed continuous-time stochastic process modeling framework, we analyzed fine-scale movements in a non-breeding population of Hudsonian godwits (*Limosa haemastica*), a gregarious long-distance migratory shorebird. Specifically, we evaluated if these extreme migrants exhibit restricted, shared, and periodic patterns of space use on one of their main non-breeding grounds in southern South America. Finally, *via* a generalized additive model, we tested if the observed patterns were consistent within a circadian cycle.

**Results:**

Overall, godwits showed finely-tuned range-residence and periodic movements (each 24–72 h), being similar between day and night. Remarkably, range-resident individuals segregated spatially into three groups. In contrast, a smaller fraction of godwits displayed unpredictable and irregular movements, adding functional connectivity within the population.

**Conclusions:**

In coastal non-breeding areas where resource availability is highly predictable due to tidal cycles, range-resident strategies during both the day and night are the common pattern in a long-distance shorebird population. Alternative patterns exhibited by a fraction of non-resident godwits provide functional connectivity and suggest that the exploratory tendency may be essential for information acquisition and associated with individual traits. The methodological approach we have used contributes to elucidate how the composition of movement phases operates during the non-breeding season in migratory species and can be replicated in non-migratory species as well. Finally, our results highlight the importance of considering movement as a continuum within the annual cycle.

**Supplementary Information:**

The online version contains supplementary material available at 10.1186/s40462-023-00411-3.

## Background

Environmental predictability has been proposed as one of the main external selective forces acting on animal movement [1, 2]. At the individual level, engrained behaviors and cognitive skills are used by mobile consumers to track phenological variation in resource availability and, consequently, reduce the uncertainty associated with environmental heterogeneity [2, 3]. In turn, depending on the spatiotemporal scale, resource tracking may lead to different movement patterns that are not mutually exclusive, such as range-residency, nomadism, and/or migration [4, 5].

In birds, nearly 19% of the extant species exhibit regular migratory movements associated with seasonal fluctuations in environmental conditions [6]. Within the annual cycle, migratory birds must synchronize different energetically demanding events — such as breeding, molting and winter survival — with favorable environmental conditions [7, 8]. As a rule, the arrival at breeding and non-breeding areas matches with periods of high resource productivity, allowing them to acquire additional energy [9]. This dynamic nature simultaneously represents an opportunity and a challenge to understand how seasonal ecological adjustments operate at different spatiotemporal scales, driving the emergence of different movement patterns throughout the life time of migratory birds [e.g., 10, 11]. For instance, over the course of their life cycle, some bird populations (or even individuals) may switch between range-residency, migration, and nomadism according to the amount of environmental variability encountered within and between years [5]. Despite this, much of the literature has focused on migration per se [12]; yet space use and movements during breeding and non-breeding season remain relatively understudied in long-distance migratory birds [13], especially at high austral latitudes.

Overall, long-distance migratory birds spend c. 6–7 months of their annual cycle in non-breeding areas [7, 14]. During this period, birds must ensure access to resources to meet their nutritional needs and cope with subsequent events of their annual cycle [15, 16]. Under these conditions, range-residency (i.e., restricted use of space) and routine movements (i.e., periodic patterns of space use) may emerge when resources are highly predictable over time [1], as they are efficient strategies to help balance the trade-off between the acquisition and conservation of energy [17]. For instance, individuals that integrate information about the locations of the most profitable patches and their temporal dynamics can reduce the time spent searching and adjust the periodicity of resource renewal rates [18]. As a result, diurnal and nocturnal circadian patterns can emerge in response to resource recovery cycles [19]. Additionally, in gregarious species, conspecifics share resources, so the use of public information during resource tracking can lead to patterns in which several individuals share common space use and movement routines [20]. Thus, routine movements within a restricted area may be advantageous for long-distance migratory birds spending the non-breeding season in predictable environments.

Here, we use GPS tracking technology and a continuous-time stochastic process (CTSP) modeling approach to investigate space use and movement patterns in a population of Hudsonian godwits (*Limosa haemastica*; hereafter, ‘godwits’) throughout their non-breeding season in Chiloé, Chile, a crucial area for this Nearctic migratory species [21]. Godwits represent a good model to explore and integrate the ideas about space use and environmental predictability. Year after year, thousands of godwits undertake long-distance journeys from Arctic-breeding areas in the northern hemisphere to non-breeding areas in the Southern Cone of South America (mainly, Chile and Argentina) and vice versa [22]. During the non-breeding season, from September to April, coastal wetlands represent critical habitats for godwit populations [23]. High-quality coastal wetlands are essential for godwits to successfully undertake future events of the annual cycle, such as their tightly scheduled northward migration and the subsequent breeding season [21, 24, 25]. In particular, evidence suggests that high austral latitude intertidal wetlands, such as those of Chiloé, may provide a predictable and abundant food supply for godwits during the non-breeding season [26]. In these coastal habitats, lunar tidal cycles periodically modulate the spatiotemporal availability of foraging and resting sites, both daily (i.e., low and high tides) and biweekly (i.e., spring and neap tides) [27]. Thus, godwits and other shorebirds must track resources that are highly predictable through space and time during these 5–7 months, oscillating daily between foraging and resting sites at low and high tides, respectively.

Likewise, the variation in the size of the area used by different individuals could be explained by morphological traits (e.g., body size) [28]. In godwits, bill length is a good proxy for body size [29]; indeed, individual differences in bill length may reflect social dominance and foraging skills, with larger individuals being generally dominant over smaller individuals [30], and thus different movement strategies to exploit resources in a tidally structured environment [e.g., 31, 32]. Outside the breeding season, godwits are tactile foragers that prey mainly on polychaete worms [33] and, as such, a longer bill allows them to access more prey, especially large worms [29].

Because godwits depend on tidal cycles to forage and rest, in response to periodic variation in the availability of resources, we predict that godwits will display temporal stability in the area used by an individual. Since they are gregarious, we also expect godwits to exhibit a restricted area use characterized by a high degree of space-use sharing and routine movements. In addition, as many shorebirds spending the non-breeding season in coastal areas, we expect godwits to forage both during the day- and night-time [34, 35]. If range-residency patterns follow tidal cycles, we expect that godwits will show regular patterns of diurnal and nocturnal activity during circadian cycles. Finally, we expect a negative relationship between the size of the area used and bill length. Individuals with longer bills (presumably dominants) will exploit resources in more restricted areas because they have access to a higher food supply and/or because they monopolize the highest quality foraging areas.

## Methods

### Study area and target population

**Fig. 1:**
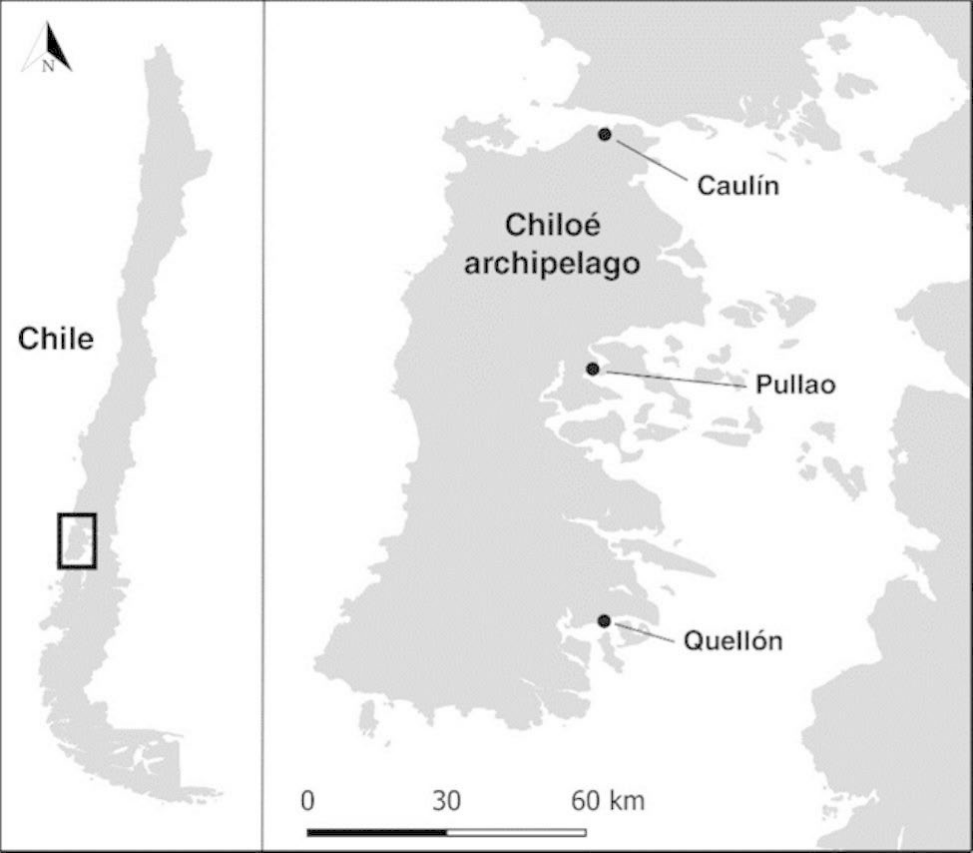
Study area in Chiloé archipelago, Chile. We focused in Caulín, Pullao and Quellón, three bays that were consistently used by godwits during non-breeding season (September to April). Map created using the Free and Open Source QGIS

This study was conducted in the Chiloé archipelago in southern Chile (≈ 42°30’ S, 73°45’ O; Fig. 1), where c. 21,000 Hudsonian godwits spend the non-breeding season [36]. The main island of the archipelago is 190 km long and 55–65 km wide [37]. Along with smaller nearby islands and several bays, it is recognized as a Site of Hemispheric Importance for the conservation of migratory shorebirds (www.whsrn.org). Godwits are distributed in three main complexes composed by different bays: Bahía de Ancud, Castro-Curaco and Huildad-Yaldad; a fourth major complex is located outside of the archipelago along the nearby mainland in the Seno de Reloncaví [see 36]. Our study area comprised three bays: Caulín, Pullao, and Quellón, each of which is located in one of the three complexes on the main island (Fig. 1).

### Capturing and GPS deployment

Godwits were captured using cannon nets during two non-breeding seasons (2016–2017 and 2017–2018) [see 38]. Each bird was weighed and its morphological structures (bill, tarsus and wing length) measured by a single person (JGN). Individuals were equipped with GPS tags (University of Amsterdam Bird Tracking System; hereafter, UvA-BiTS) [39] attached to the birds using leg-loop harnesses [40]. We tagged 20 godwits (December 2016–January 2017 and November 2017–February 2018; Table 1): nine godwits in Caulín, seven in Pullao, and four in Quellón (Fig. 1). All birds were banded with unique red coded flags and a PVC ring. We selected only adult godwits (aged following Pyle [41]), with an adequate body mass (range = 233.5–302.6 g) and in apparently good body condition. The total weight of the tag deployment (UvA-BiTS tag + harness + flag + PVC ring) was 9.8 g, i.e., 3.2–4.3% of a bird’s body mass at capture (Table 1) [42]. Sex was assigned by molecular sexing [38]. Due to their larger size and higher body mass, most tagged individuals were females (16 out of 20; Table 1). UvA-BiTS duty-cycles were programmed to record one GPS position every 30 min with a calibrated error of 3.1 m (95% CIs 2.8–3.3) [see 43].

**Table 1:** Summary of GPS tracking data along with the results of home range and periodicity analyses

ID	Flag	Sex	Sites	Equipment date	Body mass [g]	Days tracked	GPS positions	HR (CIs) [km^2^]	CA (CIs) [km^2^]	Periodicity (h)
2280	EHC	F	Caulín	04-12-2016	273.5 (3.5)	83	3451	110.69 (89.82–133.70)	23.49 (19.06–28.38)	27
2281	ELJ	M	Caulín	15-01-2017	253.2 (3.8)	98	6093	622.44 (520.16-733.75)	66.07 (55.33–77.76)	-
2289	EJA	F	Caulín	02-12-2016	249.9 (3.9)	47	1866	343.2 (318.55-368.74)	58.61 (54.40-62.98)	-
2401	JCM	F	Caulín	27-11-2017	274.4 (3.5)	71	4167	48.47 (42.89–54.39)	8.18 (7.24–9.18)	24
2402	JCV	F	Caulín	27-11-2017	242.6 (4.1)	97	5510	41.76 (37.57–46.16)	8.04 (7.23–8.89)	24
2407	JCP	F	Caulín	27-11-2017	252.8 (3.8)	45	2821	58.02 (47.90–69.10)	9.09 (7.50-10.83)	42
2408	JCX	F	Caulín	27-11-2017	233.5 (4.1)	119	6531	35.28 (32.31–38.38)	6.37 (5.83–6.93)	24
2292	EHJ	M	Caulín	04-12-2016	229.8 (4.3)	149	7062	-	-	-
2409	JCT	F	Caulín	27-11-2017	254.9 (3.8)	23	1252	54.84 (50.19–59.70)	9.24 (8.45–10.05)	36
2283	EJE	F	Pullao	02-12-2016	250.1 (3.9)	107	4432	27.56 (25.28–29.95)	3.47 (3.18–3.77)	24
2286	EJH	F	Pullao	02-12-2016	268.2 (3.6)	106	6011	49.02 (44.68–53.56)	3.82 (3.48–4.18)	72
2405	JEC	F	Pullao	29-11-2017	273 (3.5)	23	1409	172.25 (135.32-213.57)	24.57 (19.30-30.47)	29
2406	JEA	F	Pullao	29-11-2017	265 (3.6)	82	4620	998.14 (769.87-1255.59)	159.15 (122.75–200.20)	-
2412	JLA	F	Pullao	07-12-2017	264.7 (3.7)	34	2128	217.57 (169.60-271.42)	30.95 (24.12–38.61)	-
2287	EJC	F	Pullao	02-12-2016	264.1 (3.7)	112	6815	-	-	-
2291	EJM	F	Pullao	02-12-2016	263.1 (3.7)	164	7739	-	-	-
2426	JNM	F	Quellón	19-01-2018	302.6 (3.2)	60	3383	86.07 (69.73-104.09)	16.42 (13.31–19.87)	24
2427	JPJ	M	Quellón	16-02-2018	301.3 (3.2)	19	1053	248.73 (148.05–375.10)	45.38 (27.01–68.44)	-
2430	HLJ	M	Quellón	16-02-2018	271.6 (3.6)	35	2082	721.59 (404.75-1128.53)	116 (65.06-181.41)	-
2431	JME	F	Quellón	09-12-2017	271.2 (3.6)	37	2437	11.59 (8.95–14.57)	2.80 (2.16–3.52)	24

Body mass in grams [g] together with total weight (expressed as a percentage of body mass) is indicated for each individual. Home range (HR; 95% UD) and core area (CA; 50% UD) estimations are showed in square kilometers [Km^2^] with confidence intervals (CIs). Periodic patterns of space use are indicated in hours [h]. ID = identity; F = female; M = male

### Home range and core area determination

Home range and core area estimation could be negatively biased if autocorrelation in tracking data is not accounted for [44]. We used the CTSP modelling approach that accounts for autocorrelation through time of the position, velocity, or both.

We followed the workflow proposed by Calabrese et al. [45] using the R package *ctmm*. First, to determine if godwits exhibit range-residency, we started with visual verification through a variogram analysis [46]. An empirical variogram is a plot of the spatial covariance of positions as a function of the time lag between observations, enabling the evaluation of the autocorrelation structure of a tracking data set [47]. For a range-resident individual, the variogram depicts an asymptote over increasing long-lags, indicating that the tracking data set is appropriate for home range analyses [45]. We refer to range-resident individuals as those that show a restricted space use or a sedentary range. In contrast, non-resident individuals are those that do not have a sedentary range (i.e., lack of asymptote in the variogram).

Second, for resident individuals, a family of CTSP models that assume restricted space use and different autocorrelation time scales were fitted. In a nutshell, an independent identically distributed (IID) process is a null model that has no autocorrelation. The Ornstein-Uhlenbeck (OU) and the Ornstein-Uhlenbeck Foraging (OUF) processes are models that assume restricted use of space, with OU having autocorrelation only for position ($\tau$_*p*_), whereas OUF accounts for the autocorrelation of both position and velocity ($\tau$_*v*_) [46]. The best model for each individual’s tracking data was chosen by employing model selection *via* corrected Akaike Information Criterion (AIC_C_) [48]. Third, once an appropriate model was selected and fitted for each individual, we used area-corrected autocorrelated kernel density estimation (AKDE_C_) to quantify the utilization distribution (UD) with confidence intervals (CIs). AKDE_C_ yields a more accurate UD estimate, given an appropriate CTSP model that represents the autocorrelation structure of the tracking dataset [49–51]. The median area (50% UD) was used to determine the core area (i.e., the area with the highest intensity of use) [52]. Finally, to compare the results between complexes, we used the recently developed χ^2^-IG meta–analysis framework [53]. We tested for statistically significant differences between complexes through the ratio of population mean-home range areas. This ratio allows the comparison of two mean-home range areas *via* the estimation of the relevant effective sample size (N, estimated as Т/$\tau$_*p*_, where Т is the total tracking time and $\tau$_*p*_ is the positional autocorrelation parameter or home range crossing time) with CIs. Thus, for ratios with CIs which include 1, there are no statistically significant differences between complexes. However, these differences can be substantial if the CIs include values such as 1.5 or 2 [see 53].

### Home range overlap

To quantify the overlap in space used by individual godwits, we used the bias-corrected Bhattacharyya coefficient (BC) for the AKDE_C_ [54] incorporated in the package *ctmm*. BC measures the relative similarity between two UD (from 0 for no overlap to 1 for identical UDs) accompanied by an uncertainty measure *via* the estimation of CIs [55]. Following the approach proposed by Winner et al. [54], we used the CI estimates of BC to determine whether the overlap between individuals was statistically significant. Overlap measures with a minimum CI higher than 0.01 (i.e., the probability that the overlap was ≥ 0.01 is 95%) was considered significant, while a minimum CI less than 0.01 (i.e., there is no certainty that the overlap differed significantly from 0) was considered not significant. With these results, we built a weighted network for graphing the patterns and strength of interactions observed between UDs.

### Periodic patterns of space use analysis

To explore regular periodic patterns of space use in godwit populations, we followed the signal processing approach for movement data proposed by Péron et al. [56] *via* the *ctmm* framework [45]. First, we used a Lomb-Scargle periodogram (LSP) to detect the presence of periodic patterns. LSP enables visual identification of the peak frequency that makes periodic patterns apparent in movement data [56]. In order to discard artefactual periodicities created by the sampling schedule, we used a null model approach. Briefly, the CTSP model selected to estimate home range acts as the aperiodic null model. From this model, a set of simulated data is generated with the same sampling schedule as the real data. Within the simulations, periodograms of the real and simulate dataset are calculated. Finally, the p-value of the periodicity test is used to determine whether the period of interest at the periodogram peak differs significantly between the simulated and real datasets [see 56]. We then set the parameters of the periodicity test function with a null period of interest and 300 simulations. For individuals that showed a periodic pattern of space use, we fit CTSP models *via* periodic mean processes [19]. In this case, the model assumes that godwits return to a location periodically in time within a restricted area. Thus, periodic mean processes can account for the proportion of the variance in position ($\eta$_*p*_, OU and OUF) and velocity ($\eta$_*v*_, only OUF) based on the periodicity in the movement process mean [19]. Period values of the mean were specified for each individual according with the periodogram and null model approach. The most parsimonious model was selected *via* a mean square predictive error (MSPE) [57].

### Post-hoc analysis

To assess whether periodicity is related to home range size and whether this pattern is consistent between day- and night-time, post-hoc comparisons were explored by fitting a quasi-Poisson generalized additive model (GAM) with random effects *via* the *gam* function from the R package *mgcv* [58]. The model included the intensity of periodicity (i.e., $\eta$_*p*_) as a response variable, as well as home range crossing time (i.e., $\tau$_*p*_) and daily periodicity (two levels: day and night) as independent variables; identity was included as a random intercept. The smoothing terms for home range crossing time and individual variation were fitted with cubic regression splines and random effects, respectively. The smoothing parameters were estimated *via* restricted maximum likelihood estimation [58] and number of knots (k = 6) was chosen *via* model checking (*gam.check* function). As complementary method, using a continuous-time speed and distance estimation (CTSD) approach, we estimated the instantaneous speed averaged over 24-h cycles to assess the consistency in daily activity levels [59].

Finally, a simple least squares regression was used to model the relationship between home range size (km^2^) and bill length (cm). To linearize the relationship between the variables, the data were log-transformed. All analyses were performed with R version 4.2.0 [60].

## Results

### Home range and core area

**Fig. 2:**
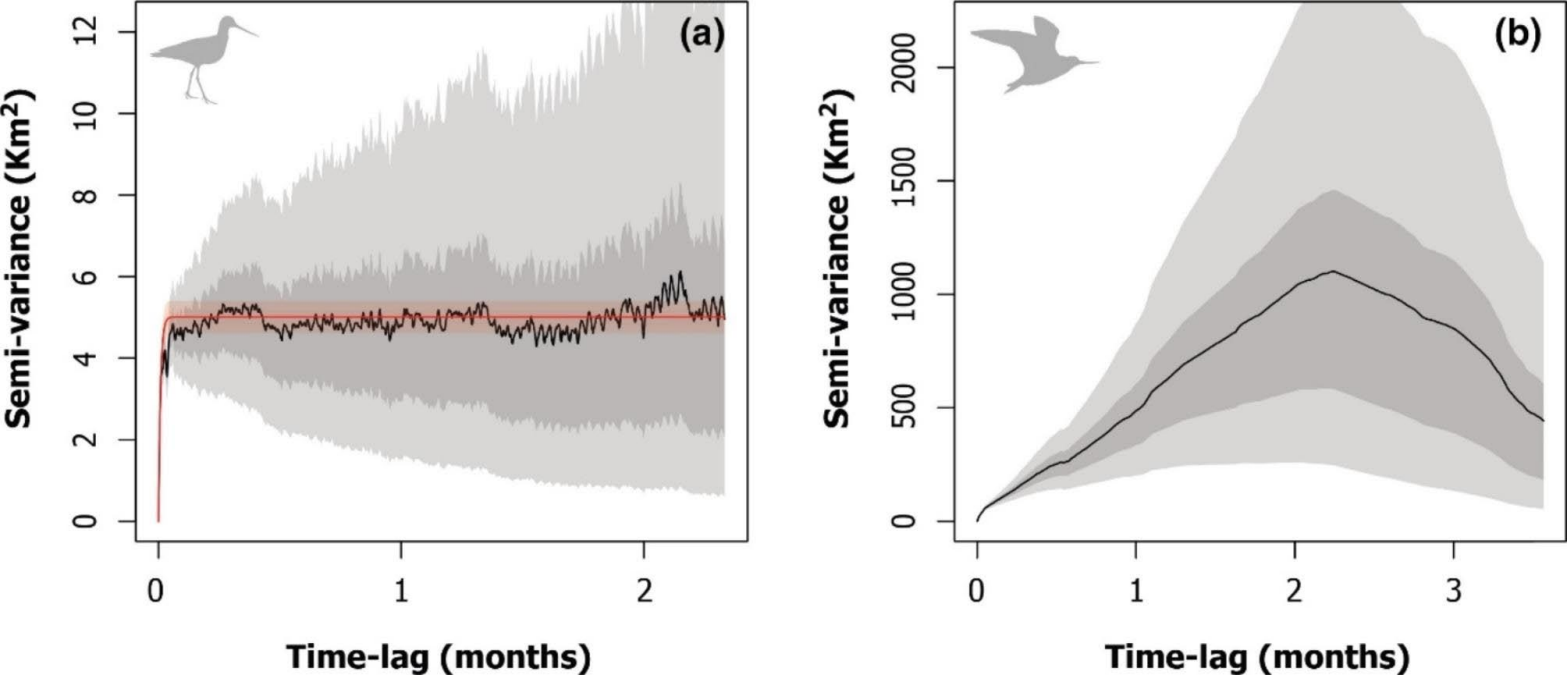
Example of range-residency verification through variogram analysis. In panel **(a)**, the empirical variogram (black line with light and dark gray shading representing 50% and 95% CIs, respectively) reaches an asymptote for longer lags, suggesting restricted use of space on a continuous timescale. The best-fit model (Ornstein-Uhlenbeck Foraging; OUF) that adequately describes the godwit movement data was fitted via theoretical variogram (red line with pink shading representing 95% CIs). In panel **(b)**, the empirical variogram does not approach an asymptote with long lags, indicating that there is no evidence of range residency. Note the different scales of both axes

**Fig. 3:**
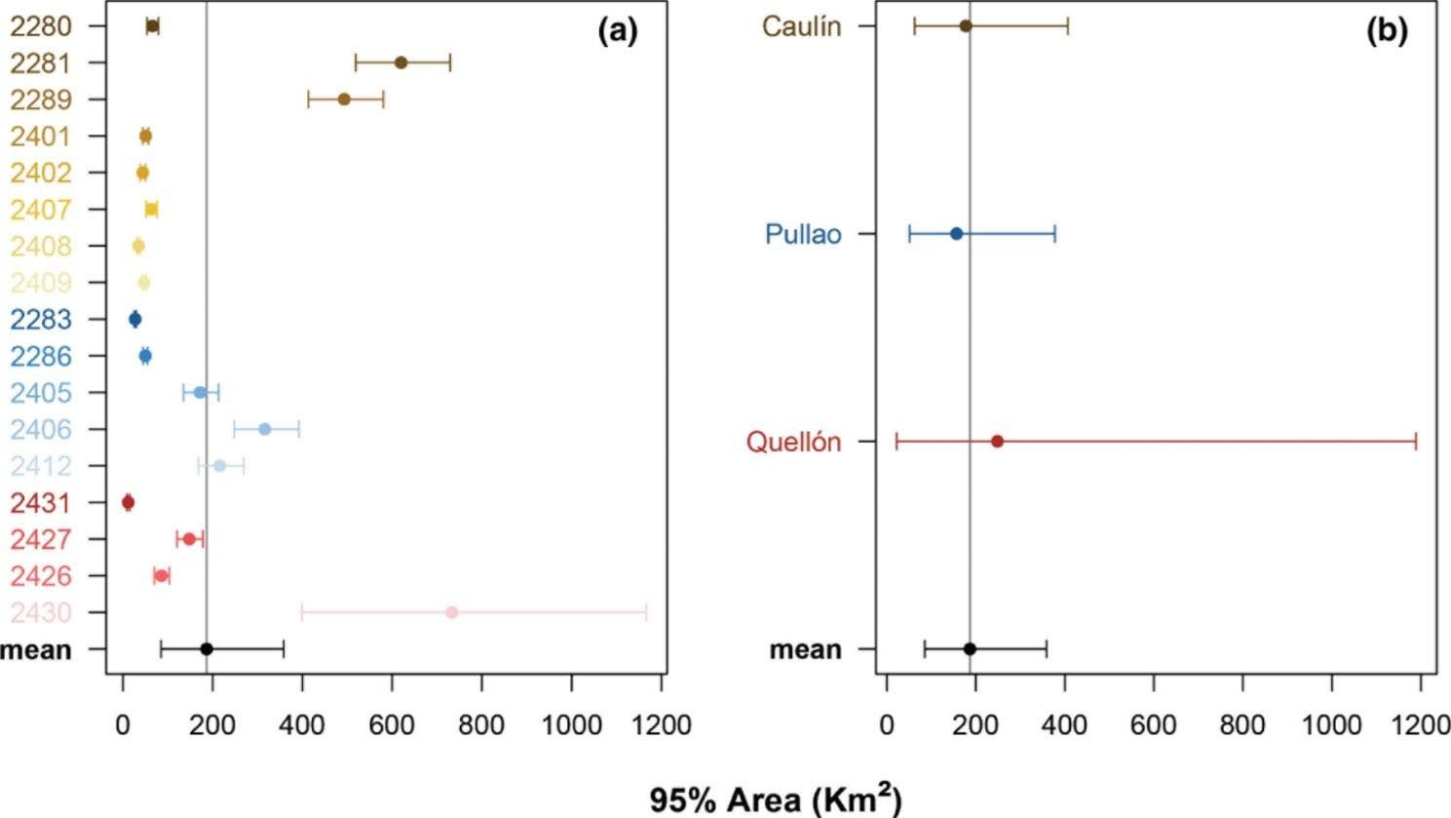
Forest plots show the relationship between individual home range **(a)** and the mean home range per bay complex **(b)** with the mean population home range area 186.52 (95% CIs 84.92–358.62; black dot) km^2^. In **(a)**, dots represent individual home range area of godwits in Caulín (brown-yellow scale), Pullao (blue scale) and Quellón (red scale). In **(b)**, dots represent the mean home range area for Caulín, Pullao and Quellón. Error bars represent the 95% CIs

**Fig. 4:**
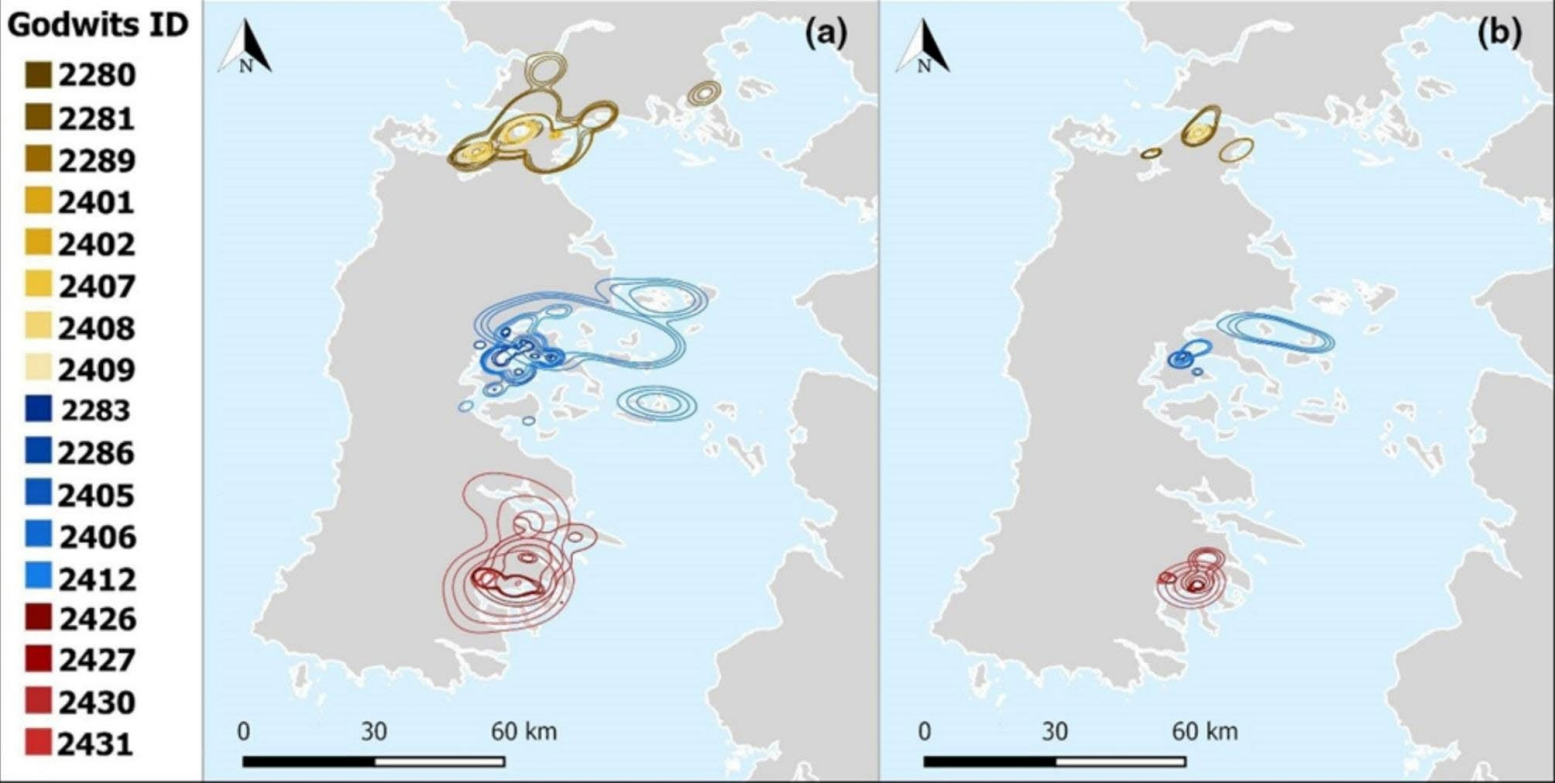
Home range (95% UD; **a**) and core area (50% UD; **b**) of godwits in Caulín (brown-yellow scale), Pullao (blue scale) and Quellón (red scale) during non-breeding season in Chiloé, Chile. For each home range and core area, the middle contour shows the UD while the inner and outer contours show the 95% CIs. Map created using the Free and Open Source QGIS

Most godwits (Caulín = 8, Pullao = 5 and Quellón = 4; *n* = 17) exhibited range-residency movements (Table 1). In contrast, the empirical variogram of three individuals, one tagged at Caulín and two at Pullao, showed no evidence of range-residency and, consequently, a home range analysis was inappropriate for them (Table 1; Fig. 2; Additional file 1: Fig. S1). Model selection favored the OUF model in all range-residency individuals when accounting for position autocorrelation, velocity autocorrelation, and restricted space use (Additional file 1: Table S1). Individual home range size varied among godwits within complexes, but mean area was similar among Caulín 177.5 (95% CIs 62.5–407.1) Km^2^, Pullao 156.7 (95% CIs 50.9–377.8) Km^2^, and Quellón 248.4 (95% CIs 22.1–1189.2) Km^2^, (Table 1; Figs. 3 and 4). Specifically, the ratios between Caulín/Pullao 0.79 (95% CIs 0.11–2.96), Quellón/Caulín 1.03 (95% CIs 0.001–5.83) and Quellón/Pullao 1.10 (95% CIs 0.001–6.32) did not show statistically significant differences. Similar patterns were observed in the core area analysis (Table 1; Fig. 4). Mean area was 26.0 (95% CIs 10.4–54.9) Km^2^ for Caulín, 20.2 (95% CIs 5.6–53.8) Km^2^ for Pullao and 40.2 (95% CIs 4.8–166.2) Km^2^ for Quellón (Additional file 1: Fig. S2). Likewise, the Caulín/Pullao, Quellón/Caulín and Quellón/Pullao ratios were 0.77 (95% CIs 0.10–3.05), 1.23 (95% CIs 0.01–5.96), and 1.19 (95% CIs 0.01– 6.35), respectively.

### Home range overlap

**Fig. 5:**
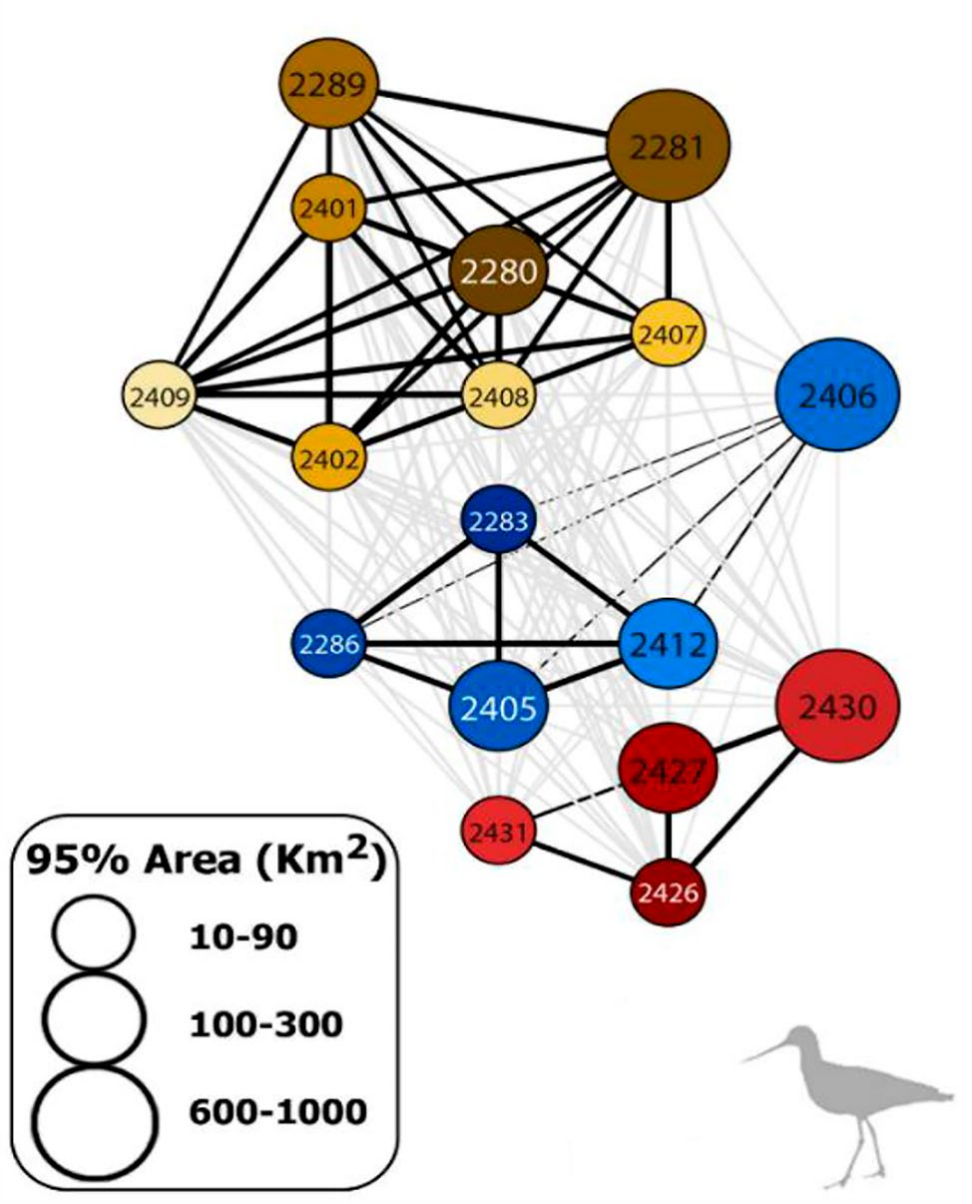
Results of Bhattacharyya coefficient (BC) via weighted network. Each edge (black lines) is associated with BC value (width of black lines is proportional to the BC value from 0.1 to 0.9). Circles with numbers represent individual home range for Caulín (brown-yellow polygons), Pullao (blue polygons) and Quellón (red polygons). Size of circles is proportional to the home range size. Finally, grey lines represent all possible paired interactions where BC is equal to 0

Range-resident individuals showed home range overlap within each complex of bays and, at the same time, a general segregation among complexes of bays (Fig. 4). BC analysis confirmed a clear clustering with significant overlap within each complex of bays and a median pairwise overlap of 0.81 (95% CIs 0.73–0.89; *n* = 28) for range-resident individuals within the northern complex of bays, 0.60 (95% CIs 0.54–0.68; *n* = 10) for the central complex, and 0.61 (95% CIs 0.43–0.72; *n* = 6) for the southern one, respectively (Fig. 5).

However, the movements of three non-resident individuals added some degree of functional connectivity between neighboring complexes (Additional file 1: Fig. S1).

### Periodic patterns of space use

The LPS indicated periodic patterns of movement in 11 out of 17 (65%) range-resident godwits (Table 1; Additional file 1: Fig. S3). Specifically, the LPS showed a strong one-day period for seven individuals and two-to-three days periods for the other four (Table 1). Notably, the non-periodic godwits also showed the largest home ranges (Table 1). The null model approach confirmed that the periodicity patterns observed were not artefactual periodicities created by sampling schedule (p = 0–0.03), suggesting a periodic pattern of c. 24–72 h, which matched with the visual diagnostic of the LPS. In all cases, the periodic mean process models with position and velocity autocorrelation (i.e., OUF) were selected, accounting for the proportion of the variance in position and velocity due to the periodicity of the mean (Additional file 1: Table S1).

### Post-hoc analysis

**Fig. 6:**
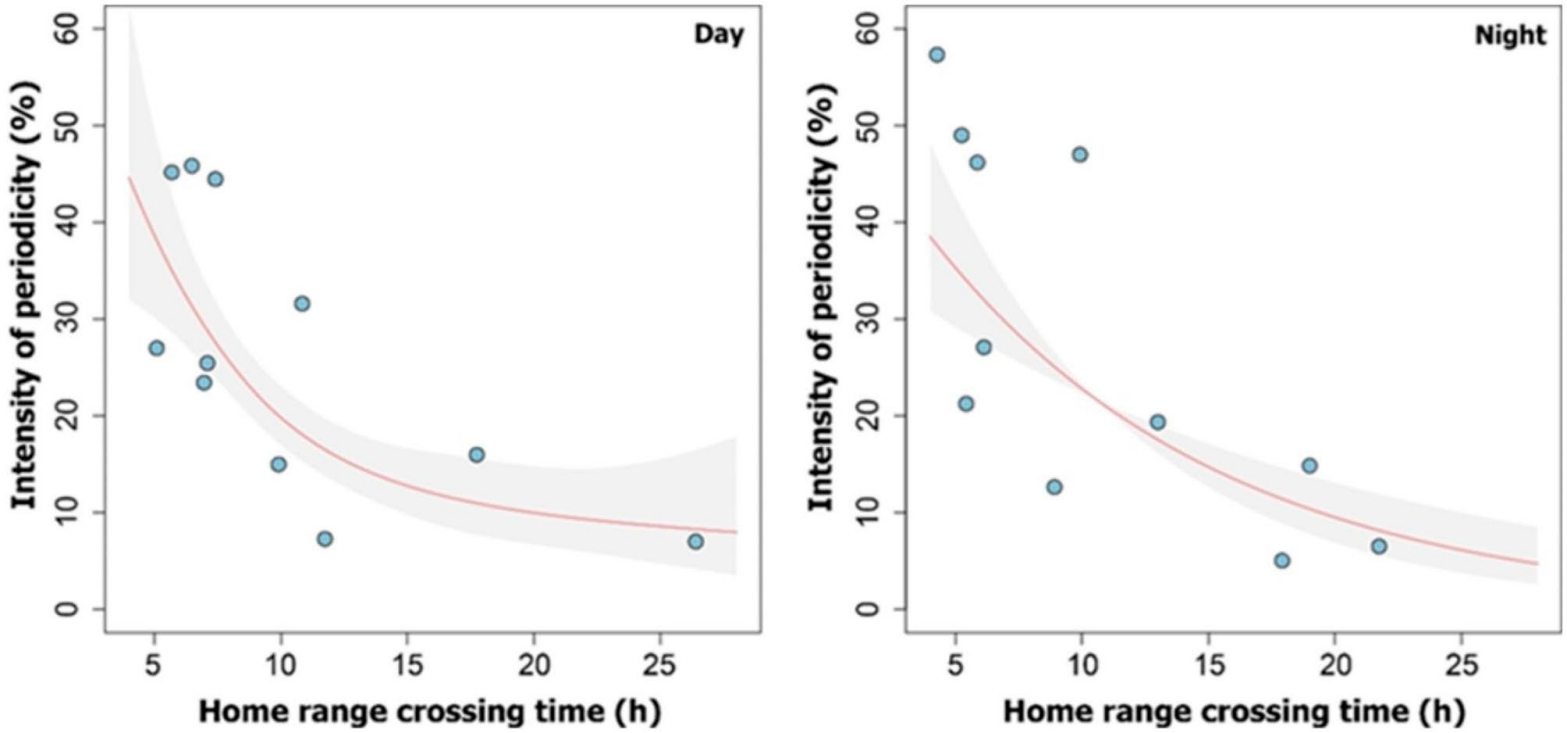
Relationship between home range crossing time and intensity of periodicity. Godwits with small home ranges revisit the same sites more frequently than those with larger home ranges. These periodic patterns of space use occur both day and night. Fitted values of non-linear smooths (red line with grey shaded areas representing 95% CIs) from the optimal generalized additive model (GAM)

A GAM analysis confirmed a significant nonlinear and decreasing relationship between intensity of periodicity and home range crossing time for both day- (p = 0.003) and night-time (p = 0.001; Fig. 6). Therefore, individuals that showed more periodic behavior also tended to move less widely within their home ranges. Likewise, CTSD estimates suggest that godwit activity levels tend to be similar between day and night (Additional file 1: Fig. S4), regardless of periodicity patterns.

The least squares regression showed a negative relationship between home range size and bill length (slope = -7.93; 95% CIs -17.98 to -2.11). However, the slope was not significant (p = 0.11) and the model fit was poor (r^2^ = 0.15).

## Discussion

Within a long-distance migratory shorebird population spending the non-breeding season in a restricted geographic area, individuals exhibited two distinct and contrasting patterns of space use. Residents - the main movement pattern in Chiloé godwits (85%) - exhibited restrictive space use characterized by gregarious and periodic movements, with this routine being similar between day and night. Remarkably, home range overlap analyses clearly show that residents are spatially segregated in three complexes of nearby bays, contrasting with non-residents (15%) that moved among complexes without settling permanently in any of them. Hence, our observed movement pathways consistently support a general spatial segregation, with non-resident individuals adding functional connectivity within the population.

Resident strategies may commonly arise in godwits tracking spatiotemporally predictable tidal cycles [1]. Via time-place learning, godwits can gain and use information that is integrated into cognitive maps [61, 62]. For instance, at a within-resource-patch scale, intensive use of core areas could result from an area-restricted search foraging strategy during low tides [63]. In turn, at a between-resource-patch scale, selection of foraging grounds also balanced a trade-off with distance to high-tide roosts [64]. Thus, the restrictive and periodic patterns exhibited by most resident godwits suggest a routine movement behavior based on the establishment of efficient routes for commuting.

As we predicted, tidal cycles lead to periodic movement patterns in range-resident godwits. In Chiloé, tides are semi-diurnal and intertidal areas are thus accessible to foraging for 5.5–6 h during low tide [23], with c. 12 h periodicity during both the day and night. In addition, lunar tidal cycles modulate the effective available foraging areas and roosting sites between spring and neap tides [27]. Hence, the strong periodicity ranging from 24 to 72 h may emerge from a balance between the availability of foraging and roosting sites, modulated by tidal amplitude [65] and with similar activity patterns between day and night. This result indicates that godwits at high austral latitudes make a complementary use of intertidal areas at night to forage in order to fulfil their daily energetic requirements, contrasting with the supplementary pattern reported for another godwit species that also forage at night in coastal temperate areas to supplement the energy not acquired during the day [34, 66].

Strong home range overlap within each bay complex also suggests that shared space-used and segregation could be the result of public information use and diplomacy among godwits, two mutually non-exclusive explanations. Public information use as a positive proximate cue for selecting suitable habitats can lead to aggregation in areas with high conspecific density within a fragmented landscape [e.g., 67, 68]. Under a diplomacy scenario, spatial segregation would avoid interference competition for resources between conspecifics from different groups [69]. Both in marine and terrestrial birds that breed colonially, evidence suggests that central-place foragers establish colony-specific foraging areas that do not overlap, mitigating intraspecific competition with neighboring colonies [e.g., 69–71]. Godwit species are gregarious animals that forage and rest in tight groups [72, 73]. Therefore, they must trade-off benefits (e.g., enhance their chances of foraging success and anti-predator defense) and costs (e.g., interference competition, predator attraction) of group living [74, 75].

Similar results have been reported for other godwit species, suggesting that populations are composed by resident individuals with range restricted patterns of space use. For instance, while staging in rice fields in southwestern Iberia, Black-tailed godwits (*Limosa limosa*) exhibited small home ranges with core areas centered on roosting sites to avoid long foraging movements and, consequently, reduce energy expenditure [76]. Likewise, evidence suggests that during the non-breeding season in coastal areas, the home range and core area of Bar-tailed godwits (*Limosa lapponica*) was determined by short distances between foraging and roosting sites [77]. However, our results also contrast with those reported for other shorebird species in non-breeding areas. For instance, in Red knots (*Calidris canutus*) site fidelity (“solitary residents”) and aggregation (“grouping nomads”) are different strategies that respond to the distribution of resources and predation risk [78, 79]. This is not the case for resident godwits in Chiloé, where individuals exhibiting restricted area use demonstrated both strong site fidelity and high home range overlap between segregated groups. We suggest that the movement patterns observed in range-resident godwits could emerge as a habitat selection strategy to reduce costs related to search effort, settlement, and competition, and to indirectly assess habitat quality [68] within complexes of nearby bays with different levels of fragmentation and human pressure [23, 36].

On the other hand, according to the analysis of the variograms the patterns shown by the non-resident godwits corresponded with nomadic movements [5, 46]. Nomadism is expected to emerge in response to environmental variability and unpredictability [4]. However, due to the high predictability of tidal cycles, fleetingness of resources does not seem to be the main driver of nomadic movements in non-resident godwits. Alternatively, the pattern could be the result of a trade-off between the risks and rewards of information acquisition, as well as alternating periods of residency with exploratory movements [5]. Some individual traits such as body condition, experience (e.g., age), or personality (e.g., boldness) can lead to an exploratory tendency scaling to nomadic patterns [80, 81]. In this light, non-resident godwits may move irregularly over time but at the same time prospect and revisit known profitable areas. Functional connectivity between groups suggest that non-resident godwits could respond to aggregation of resident godwits displaying periods of restrictive area use [82]. In addition, two out of three non-resident godwits later spent 5 to 6 months oversummering in inland wetlands of Argentina [83], and the third surprisingly traveled to the main South American continent for ten days before returning to Chiloé for the boreal summer. These irregular patterns were consistent in at least one individual during the following non-breeding season at Chiloé. Therefore, non-resident godwits could move between temporary settlement areas where exploratory movements between known and unknown sites may be essential for information acquisition [84]. Nevertheless, inference about non-resident godwits in our study is limited and further research is required.

Contrary to previous evidence in other godwit species [e.g., 31], our prediction about home range size and bill length was not supported by the data. This could be explained by the high availability of polychaete worms (the preferred prey items of godwits) on Chiloé [26, 33], as well as a general low rate of interspecific competition in high austral latitudes. In this scenario, godwits with different bill lengths may be able to successfully exploit the rich food supply in the same bays by minimizing the number of patches visited and, consequently, having similar home range sizes. Godwits do not exhibit sexual segregation within Chiloé [38] and non-adult individuals seem to be uncommon during the non-breeding season (JGN unpublished observations) [37]. However, we recognize that the little variation in bill size (i.e., most individuals were females) could have masked a possible significant effect. In addition, our findings were derived from adults, thus potentially hampering extrapolation to the entire population. Finally, while we cannot discard the potential effect of the transmitters on individual movements [85], the consistency of our results suggests that godwits experienced no evident handicap affecting their movement behavior. Therefore, considering that we equipped godwits across all of Chiloé, we remain confident that our findings are representative of overall movement patterns exhibited by the godwit population spending the non-breeding season in coastal areas at high austral latitudes.

## Conclusion

According to our results, godwits at high austral latitudes exhibited finely-tuned space use and movement patterns that may be modulated by predictable resource availability derived from tidal cycles. More broadly, our results contribute to the understanding of how the composition of movement phases operates during the non-breeding season in a long-distance migratory species and, consequently, can be integrated into their lifetime movement patterns [10]. We integrated a continuous-time movement modeling framework that can have a broad scope and, therefore, be replicated in different ecological and conservation contexts, with either migratory or non-migratory species. Future analyses of long-term tracking data considering individual-level factors can be relevant for understanding drivers of resident and non-resident movements in long-distance migratory shorebird populations during the non-breeding season. Such studies can also reveal whether non-adult individuals follow movement patterns similar to those of adults or experience some type of nomadism (e.g., phase nomadism) [5]. Likewise, a larger sample size of both sexes is necessary to assess whether morphological traits like bill size affects space use as well. In addition, analyses of movement attributes -and how these underlie the spatiotemporal availability of resources that our data do not allow us to explore in detail- may be relevant to establish habitat preferences along with the identification of routes that are key to maintain functional connectivity -at a between-resource-patch scale [86].

### Electronic supplementary material

Below is the link to the electronic supplementary material.

Supplementary Material 1: Supplementary results for “GPS tracking analyses reveal finely-tuned shorebird space use and movement patterns throughout the non-breeding season in high-latitude austral intertidal areas”. Table and figures that complement the results of the main text.

## Data Availability

The datasets generated during the current study are available in The Virtual Lab for Bird Movement Modelling repository (https://www.uva-bits.nl/virtual-lab/) upon request to JGN (jgnavedo@uach.cl).

## Abbreviations

AIC_C_: Corrected Akaike Information Criterion
AKDE_C_: Area-corrected Autocorrelated Kernel Density Estimation
BC: Bhattacharyya Coefficient
CIs: Confidence Intervals
CTSD: Continuous-Time Speed and Distance
CTSP: Continuous-Time Stochastic Process
GAM: Generalized Additive Model
GPS: Global Positioning System
IID: Independent Identically Distributed
KDEs: Kernel Density Estimators
LSP: Lomb-Scargle Periodogram
MCP: Minimum Convex Polygon
MSPE: Mean Square Predictive Error
OU: Ornstein-Uhlenbeck
OUF: Ornstein-Uhlenbeck Foraging
UD: Utilization Distribution
UvA-BiTS: University of Amsterdam Bird Tracking System
