# Supplementary material for: GPS tracking analyses reveal finely-tuned shorebird space use and movement patterns throughout the non-breeding season in high-latitude austral intertidal areas
Source: Mov Ecol. 2023 Sep 1;11:55. doi: 10.1186/s40462-023-00411-3 (PMC10474677; doi:10.1186/s40462-023-00411-3)
Supplement: Supplementary file 1 — Supplementary Material 1: Supplementary results for “GPS tracking analyses reveal finely-tuned shorebird space use and movement patterns throughout the non-breeding season in high-latitude austral intertidal areas”. Table and figures that complement the results of the main text. [file 40462_2023_411_MOESM1_ESM.docx]

**Supplementary results for “GPS tracking analyses reveal finely-tuned shorebird space use and movement patterns throughout the non-breeding season in high-latitude austral intertidal areas”**

Enzo Basso^1,2^, Johannes Horstmann^1^, Eldar Rakhimberdiev^3^, José M. Abad-Gómez^4^, José A. Masero^5^, Jorge S. Gutiérrez^5^, Jorge Valenzuela^6^, Jorge Ruiz^1,7^ & Juan G. Navedo^1,4,7,8^

^1^ Bird Ecology Lab, Instituto de Ciencias Marinas y Limnológicas, Universidad Austral de Chile, Valdivia, Chile.

^2^ Programa de Doctorado en Ecología y Evolución, Facultad de Ciencias, Universidad Austral de Chile, Valdivia, Chile.

^3^ Institute for Biodiversity and Ecosystem Dynamics, University of Amsterdam, Amsterdam, The Netherlands.

^4^ Department of Anatomy, Cell Biology and Zoology, Faculty of Sciences, University of Extremadura, Badajoz, Spain.

^5^ Ecology in the Anthropocene, Associated Unit CSIC-UEX, Zoology, Faculty of Sciences, University of Extremadura, Badajoz, Spain.

^6^ Centro de Estudios y Conservación del Patrimonio Natural (CECPAN), Chiloé, Chile.

^7^ Estación Experimental Quempillén, Facultad de Ciencias, Universidad Austral de Chile, Chiloé, Chile.

^8^ Millennium Institute Biodiversity of Antarctic and Subantarctic Ecosystems (BASE), Santiago, Chile.

**Table S1.** Output of the continuous-time stochastic process models with stationary mean (CTSP_S_) and periodic mean (CTSP_P_)

| **ID** | **Sites** | **CTSP_S_** | $\boldsymbol{\tau}\mathbf{p}$ ***(CIs) [h]*** | $\boldsymbol{\tau}\mathbf{v}$ ***(CIs) [min]*** | **CTSP_P_**  $\boldsymbol{\eta}\mathbf{p}$ ***(CIs) [%]*** |  | $\boldsymbol{\eta}\mathbf{v}$ ***(CIs) [%]*** |
| --- | --- | --- | --- | --- | --- | --- | --- |
| 2280 | Caulín | OUF | 20.3 (16.1-25.6) | 3.5 (1.9-6.7) | OUF | 6.9 (5.9-8.2) | 0.3 (0.2-0.5) |
| 2281 | Caulín | OUF | 17.1 (14.2-20.8) | 4.1 (3.6-4.4) | ­- | ­- | ­- |
| 2289 | Caulín | OUF | 23.1 (15.5-34.4) | 12.6 (6.8-23.4) | ­- | ­- | ­- |
| 2401 | Caulín | OUF | 5.5 (4.8-6.4) | 10.7 (9.7-11.8) | OUF | 47.8 (41.4-55.3) | 8.1 (6.6-9.9) |
| 2402 | Caulín | OUF | 5.8 (5.2-6.6) | 6.5 (5.6-7.7) | OUF | 47 (41.8-52.8) | 4.1 (2-8.2) |
| 2407 | Caulín | OUF | 8.7 (7-10.9) | 5.4 (3.8-7.6) | OUF | 41.3 (22.6-75.2) | 4.1 (2.4-6.6) |
| 2408 | Caulín | OUF | 5.3 (4.8-5.8) | 3.2 (2.8-3.7) | OUF | 49.5 (45.2-54.3) | 4.9 (3.7-6.4) |
| 2409 | Caulín | OUF | 5.3 (4.8-5.8) | 3.5 (3.1-4) | OUF | 14.2 (7.7-20.2) | 7.2 (1.9-7.6) |
| 2283 | Pullao | OUF | 4.6 (4.1-5.1) | 2.6 (1.4-5.1) | OUF | 11.8 (6.5-21.2) | 1.8 (0.7-4.5) |
| 2286 | Pullao | OUF | 5.3 (4.8-5.9) | 1.9 (1.4-2.6) | OUF | 27.4 (17.5-42.8) | 1.4 (0.5-3.9) |
| 2405 | Pullao | OUF | 7 (5.3-9.2) | 2.4 (1.5-3.9) | OUF | 22.7 (11-46.7) | 6.5 (3.6-11.7) |
| 2406 | Pullao | OUF | 30.2 (22.8-40) | 3.2 (2.7-3.7) | ­- | ­- | ­- |
| 2412 | Pullao | OUF | 11 (8.3-14.5) | 4.2 (3.2-5.6) | ­- | ­- | ­- |
| 2426 | Quellón | OUF | 14.2 (11.3-17.9) | 1.3 (0.4-3.9) | OUF | 6.1 (5.1-7.3) | 0.4 (0.2-0.8) |
| 2427 | Quellón | OUF | 21.9 (11.5-41.7) | 16.1 (13.2-19.6) | ­- | ­- | ­- |
| 2430 | Quellón | OUF | 55.7 (26.2-118.7) | 2.3 (1.1-4.9) | ­- | ­- | ­- |
| 2431 | Quellón | OUF | 12 (8.8-16.4) | 1.5 (0.8-3.1) | OUF | 38.1 (29.4-49.5) | 5.3 (2-13.8) |

Ornstein-Uhlenbeck Foraging (OUF) process was selected for home range estimation (CTSP_S_) and periodicity analyses (CTSP_P_). In CTSP_S_, the timescales in position and velocity autocorrelation are described by tau position ($\tau$_p_) and tau velocity ($\tau$_v_). In CTSP_P_, the proportion of variance in position and velocity that is explained by the periodicity in the movement mean is described by eta position ($\eta$_p_) and eta velocity ($\eta$_v_), respectively. ID = identity; CIs = confidence intervals.


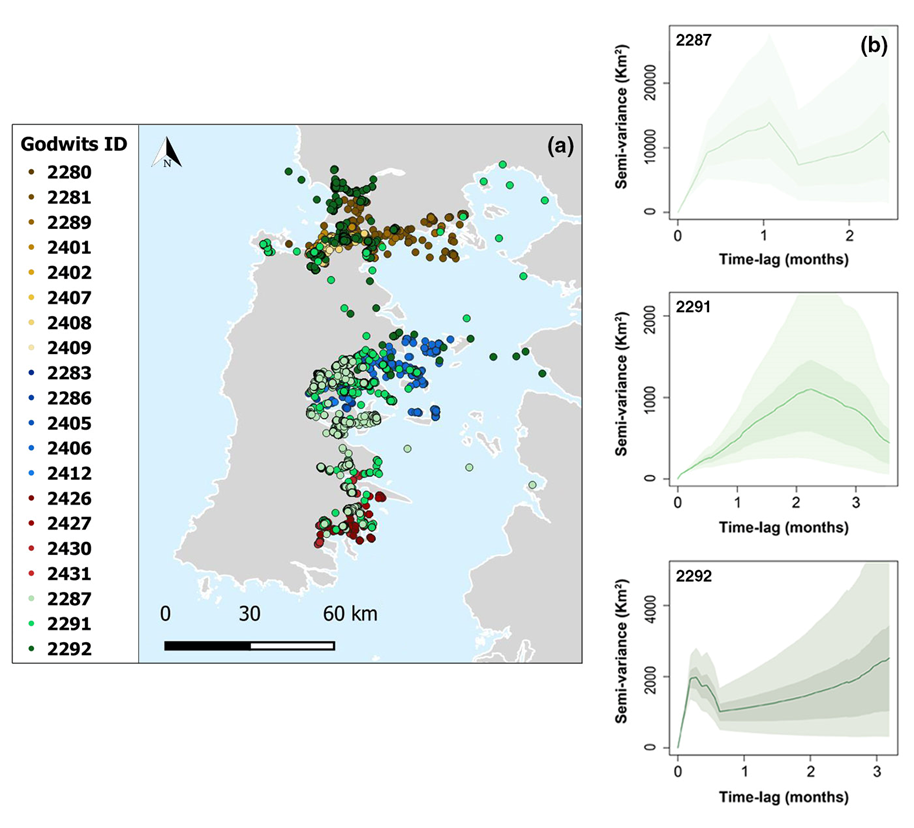


**Figure S1.** GPS locations for 20 godwits tracked in Chiloé archipelago **(a)** Range-resident godwits are coloured with brown-yellow dots for Caulín, blue dots for Pullao and red dots for Quellón. Non-resident godwits are coloured with green dots and move among the complexes of bays and make trips outside of the archipelago. These exploratory movements are reflected in the variograms **(b)** that show the absence of a clear asymptote during the tracking period. Note the different scales of axes. Map created using the Free and Open Source QGIS.


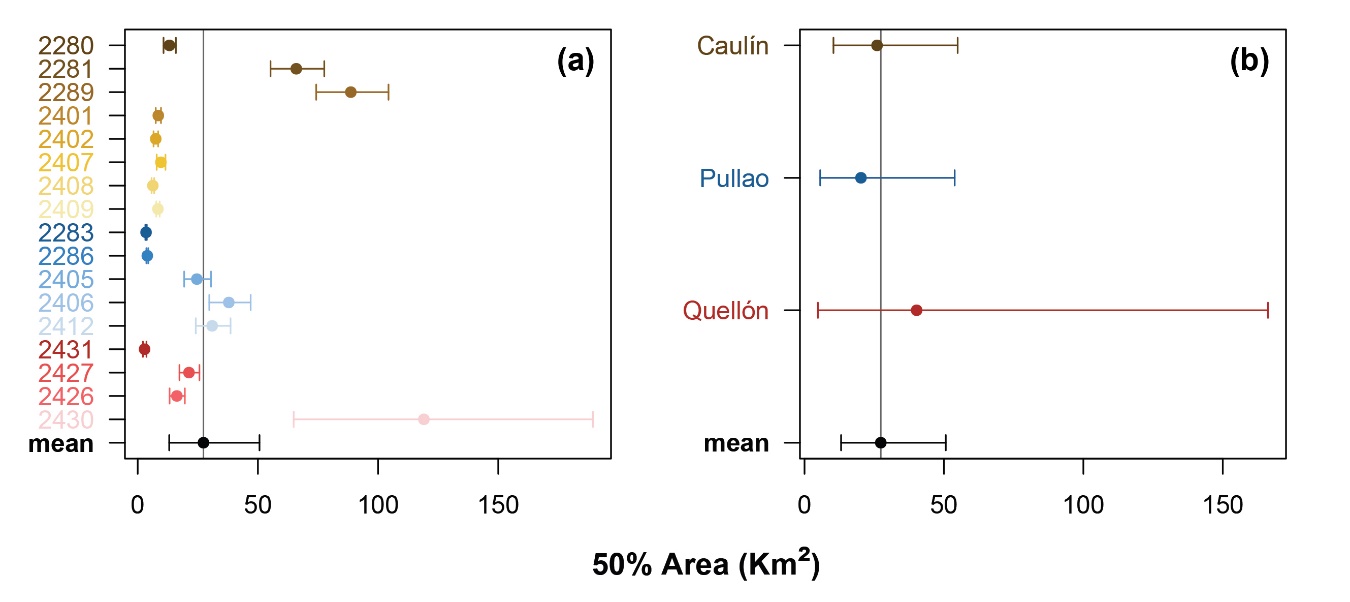


**Figure S2.** Forest plots show the relationship between individual core area of godwits **(a)** and the mean core area per complex of bays within Chiloé archipelago **(b)** with the mean population core area being 27.35 (95% CIs 13.11–50.65; black dot) Km^2^. In **(a)**, dots represent individual core area of godwits in Caulín (brown-yellow scale), Pullao (blue scale) and Quellón (red scale). In **(b)**, dots represent the mean core area for Caulín, Pullao and Quellón. Error bars represent the 95% CIs.


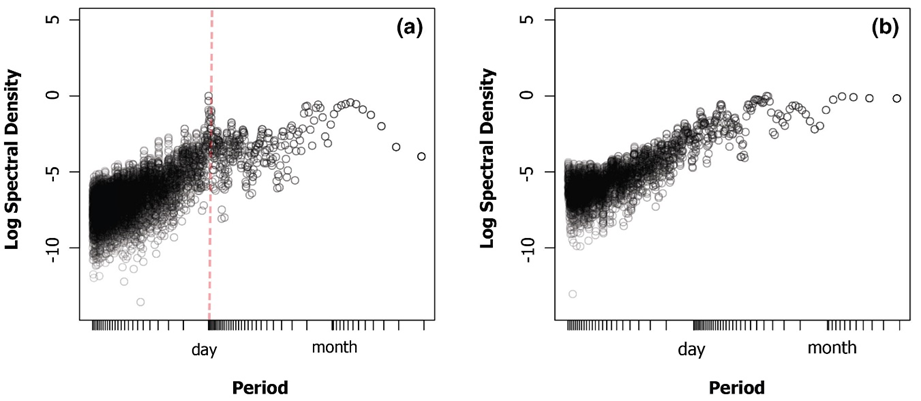
**Figure S3.** Example of visual diagnostic of periodicity patterns in godwits locations within Chiloé archipelago. Lomb-Scargle periodogram (LPS) show two different scenarios. In panel **(a)**, LPS reach a peak in one day (dashed red vertical line), suggesting a periodic pattern of c. 24 h. In panel **(b)**, the absence of a peak is indicative of no signal of periodicity in location (i.e., no evidence of periodic pattern in space use).

**
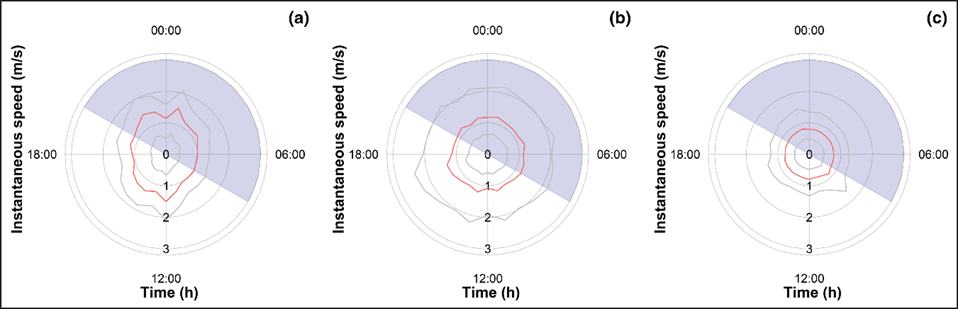
**

**Figure S4.** Example of the instantaneous speed estimation (CTSD), averaged over 24 h cycles. Radial plots depict the instantaneous speed (red line) with 95% CIs (grey lines) for the individuals 2401 at Caulín **(a)**, 2412 at Pullao **(b)** and 2431 at Quellón **(c)**. Shading in panels represent nighttime in Chiloé.
